# *Helicobacter pylori* infection downregulates duodenal CFTR and SLC26A6 expressions through TGFβ signaling pathway

**DOI:** 10.1186/s12866-018-1230-8

**Published:** 2018-08-17

**Authors:** Guorong Wen, Shili Deng, Wenfeng Song, Hai Jin, Jingyu Xu, Xuemei Liu, Rui Xie, Penghong Song, Biguang Tuo

**Affiliations:** 1 Department of Gastroenterology, Affiliated Hospital, Zunyi Medical College, 149 Dalian Road, Zunyi, 563003 China; 2 Digestive Disease Institute of Guizhou Province, Zunyi, China; 3 Clinical Medical Research Center of Guizhou Province for Digestive Diseases, Zunyi, China; 4 Key Laboratory of Combined Multi-organ Transplantation, Ministry of Public Health, First Affiliated Hospital, School of Medicine, Zhejiang University, Hangzhou, China; 5 Collaborative innovation center for Diagnosis treatment of infectious diseases, First Affiliated Hospital, School of Medicine, Zhejiang University, Hangzhou, China

**Keywords:** Duodenal ulcer, *Helicobacter pylori*, CFTR, SLC26A6, TGFβ

## Abstract

**Background:**

The pathogenesis of *Helicobacter pylori* (*H. pylori)* infection-induced duodenal ulcer remains to be elucidated. Duodenal mucosal bicarbonate secretion is the most important protective factor against acid-induced mucosal injury. We previously revealed that *H. pylori* infection downregulated the expression and functional activity of duodenal mucosal cystic fibrosis transmembrane conductance regulator (CFTR) and solute linked carrier 26 gene family A6 (SLC26A6) which are the two key duodenal mucosal epithelial cellular bicarbonate transporters to mediate duodenal bicarbonate secretion. In this study, we investigated the mechanism of *H. pylori* infection-induced duodenal CFTR and SLC26A6 expression downregulation.

**Results:**

We found that *H. pylori* infection induced the increase of serum transforming growth factor β (TGFβ) level and duodenal mucosal TGFβ expression and the decrease of duodenal mucosal CFTR and SLC26A6 expressions in C57 BL/6 mice. The results from the experiments of human duodenal epithelial cells (SCBN) showed that *H. pylori* increased TGFβ production and decreased CFTR and SLC26A6 expressions in SCBN cells. TGFβ inhibitor SB431542 reversed the *H. pylori-*induced CFTR and SLC26A6 expression decreases. The further results showed that TGFβ directly decreased CFTR and SLC26A6 expressions in SCBN cells. TGFβ induced the phosphorylation of p38 mitogen-activated protein kinase (MAPK) and P38 MAPK inhibitor SB203580 reversed the TGFβ-induced CFTR and SLC26A6 expression decreases.

**Conclusions:**

*H. pylori* infection downregulates duodenal epithelial cellular CFTR and SLC26A6 expressions through TGFβ-mediated P38 MAPK signaling pathway, which contributes to further elucidating the pathogenesis of *H. pylori-*associated duodenal ulcer.

## Background

Duodenal ulcer is a common disease in the digestive tract [1, 2]. It has been demonstrated that *Helicobacter pylori* (*H. pylori*) infection is main etiologic agent responsible for duodenal ulcerogenesis [1, 3, 4]. In spite of extensive studies, the pathogenesis of *H. pylori* infection-induced duodenal ulcer remains to be elucidated.

Duodenal mucosal bicarbonate secretion is the most important protective factor against acid-induced duodenal mucosal injury [5, 6]. A clinical study showed that there was significant diminished duodenal mucosal bicarbonate secretion in the patients with *H. pylori*-associated duodenal ulcer in comparison with healthy controls, and duodenal mucosal bicarbonate secretion returned to normal levels after the eradication of *H. pylori* [7]. The studies from animal experiments showed that intraluminal perfusion of *H. pylori* water extract inhibited acid-stimulated duodenal mucosal bicarbonate secretion in rats [8]. Prostaglandin E2 (PGE2)-stimulated murine duodenal mucosal bicarbonate secretion in vitro was also strongly inhibited by water extract from cytotoxin-associated gen A (CagA) /vacuolating cytotoxin A (VacA)-positive *H. pylori* strains [9]. These studies suggest that the effect of *H. pylori* on duodenal mucosal bicarbonate secretion might be involved in the pathogenesis of *H. pylori*-associated duodenal ulcer. However, the mechanisms whereby *H. pylori* influences duodenal mucosal bicarbonate secretion are not completely understood.

Duodenal mucosal bicarbonate secretion is mediated by bicarbonate transporting proteins located in duodenal mucosal epithelial cells. The cystic fibrosis transmembrane conductance regulator (CFTR) and solute linked carrier 26 gene family A6 (SLC26A6) are the two key bicarbonate transporting proteins of duodenal mucosal epithelial cells and they play important role in the regulation of duodenal mucosal bicarbonate secretion [10–12]. We previously showed that *H. pylori* infection downregulated the expression and functional activity of duodenal mucosal CFTR and SLC26A6 [13], which contributes to the development of duodenal ulcer. In this study, we investigated the mechanism of *H. pylori* infection-induced duodenal CFTR and SLC26A6 expression downregulation. We hope to further elucidate the mechanisms whereby *H. pylori* infection influences duodenal mucosal HCO_3_^−^ secretion and the pathogenesis of *H. pylori* infection*-*induced duodenal ulcer.

## Methods

### Reagents

Prostaglandin E_2_ (PGE_2_) and forskolin were purchased from Sigma. pH-sensitive fluorescent dye, 2′,7′-bis(2-carboxyethyl)-5(6)-carboxy-fluorescein acetoxymethyl ester (BCECF-AM), was from Invitrogen. Anti-CFTR, anti-SLC26A6, anti-P38, anti-phospho-P38, and anti-β-actin antibodies were from Santa Cruz. All other chemicals in solutions were obtained from Sigma and Calbiochem.

### *H. pylori* strain

*H. pylori* strain (ATCC 43504), from the *H. pylori* Strain Pool, Beijing, China, was verified to be cytotoxin-associated gen A (CagA)- and vacuolating cytotoxin A (VacA)-positive previously [14] and used throughout the experiments. The CagA and VacA s1/s2 and m1/m2 genotypes of the *H. pylori* strain were further confirmed by specific polymerase chain reaction (PCR) as described previously by Miernyk et al. [15]. The *H. pylori* strain was routinely cultured for 48 h on Brucella agar plates containing 5% sheep blood at 37 °C under microaerophilic conditions in a humidified CO_2_ incubator (Thermo Fisher Scientific, Wilmington, DE) and then used for experiments.

### *H. pylori* infection of mice

Male C57 BL/6 mice of 6 to 8 weeks from Shanghai Animal Center (Chinese Academy of Science, Shanghai, China) were used in this study. The animal experiments were approved by the Experimental Animal Ethics Committee of Zunyi Medical College and conducted in accordance with principles stated in the Guide for the Care and Use of Laboratory Animals (NIH publication 8623, National Institutes of Health, Bethesda, MD, 1985). The mice were housed in the experimental animal facility with specific pathogen free (SPF) of Zunyi Medical College under standard care conditions. 60 mice were used for *H. pylori* infection experiments and 10 mice were used as controls. After a week of acclimatization, the mice were orally gavaged with 0.5 ml *H. pylori* suspension in Brucella broth (1 × 10^9^ CFU/ml) once daily for 4 consecutive days. The control mice were only gavaged with sterile Brucella broth. The mice were sacrificed by cervical dislocation under CO_2_ narcosis at 4 week after last gavage. Blood samples were quickly obtained by cardiac puncture for serum transforming growth factor β (TGFβ) level examination. The gastric antrum tissues were used for *H. pylori* infection examination. The duodenal mucosal tissues were used for examination of CFTR and SLC26A6 mRNA and protein expressions and TGFβ mRNA expression.

The presence of *H. pylori* in the stomach was determined by Giemsa-stained sections. *H*. *pylori-*negative was defined when Giemsa staining was determined to be negative by visual observation, and *H*. *pylori*-positive was defined when positive Giemsa staining was observed. *H. pylori* infection density was assessed by semiquantitative analysis of *H*. *pylori* levels in the gastric mucosa (+, less than 10 bacteria per gland; ++, 10 to 20 bacteria in at least one gland; +++, 20 to 30 bacteria in at least one gland; ++++, more than 30 bacteria in at least one gland).

### *H. pylori* infection of duodenal epithelial cells

SCBN (a gift from Dr. Hui Dong in University of California San Diego) is a nontumorigenic duodenal epithelial cell line obtained from a human patient [16]. SCBN cells were maintained in Dulbecco’s modified Eagle’s medium (DMEM) supplemented with 10% fetal calf serum and 50 mg/ml penicillin-streptomycin for two days. Then 5 × 10^6^ SCBN cells were seeded on six-well plates and grown at 37 °C in a 5% CO_2_ atmosphere. Prior to infection, each well was washed twice in 1 ml of antibiotic-free cell culture medium. Then *H. pylori* was added to the cultured cells at different multiplicity of infection (MOI). After incubation for 24 h at 37 °C in a 5% CO_2_ atmosphere, the cells were harvested for examination of CFTR and SLC26A6 protein expression levels. The supernatants were for examination of TGFβ concentration. For intracellular pH measurement, SCBN cells were grown on 12-mm round coverslips and then incubated with *H. pylori* at a MOI value of 400 for 24 h. In addition, an uninfected control was included in each experiment.

### Measurement of TGFβ concentration

For the measurement of serum TGFβ concentration, after blood was quickly obtained by cardiac puncture, the blood sample was centrifuged immediately at 3000 rpm for 5 min for collection of serum. The serum sample was stored at − 20 °C and analyzed within one week. Serum TGFβ level was detected by using the enzyme-linked immunosorbent assay kit (ELISA) (TGFβ1 Mouse Uncoated ELISA Kit, Invitrogen, USA, Cat. No. 88–50,690-22) according to the manufacturer’s instructions. TGFβ level in supernatant of *H. pylori*-infected SCBN cells was detected by using TGFβ1 Human Uncoated ELISA Kit (Invitrogen, USA, Cat. No. 88–50,390-77).

### Quantitative real-time reverse transcription PCR analysis for mRNA expressions of CFTR, SLC26A6, and TGFβ

Total RNA extract of murine duodenal mucosal tissues and quantitative real-time reverse transcription PCR analysis were performed as described previously [17]. The mRNA expression level of CFTR, SLC26A6, or TGFβ was normalized to that of β-actin and was expressed as a ratio relative to β-actin. The primers were as follows: CFTR, forward 5′-AAGGCGGCCTATATGAGGTT-3′ and reverse 5′-AGGACGATTCCGTTGATGAC-3′; SLC26A6, forward 5′-GGTGGTGAAGCTGTTGAATGAC-3′ and reverse 5′-ATGTTGCCCACGACATCTACCTC-3′; TGFβ, forward 5′-ATACGCCTGAGTGGCTGTC-3′ and reverse 5′-GCCCTGTATTCCGTCTCCT-3′; β-actin, forward 5′-CTGCCTGACGGCCAAGTC-3′ and reverse 5′-CAAGAAGGAAGGCTGGAAAAGA-3′.

### Western blot analysis for CFTR, SLC26A6, P38, and Phospho-P38 expressions

Murine duodenal mucosal tissues or SCBN cells were homogenized in lysis buffer at 4 °C and western blot analysis was performed as described previously [18]. Anti-CFTR, anti-SLC26A6, anti-P38, anti-Phospho-P38, or anti-β-actin (served as internal control) was used as primary antibody. The results were expressed as the ratio relative to β-actin.

### Measurement of duodenal epithelial cellular bicarbonate secretion

Bicarbonate secretion in SCBN cells was determined through the measurement of intracellular pH [pHi] by using pH-sensitive fluorescent dye BCECF-AM as described previously [19]. When forskolin or PGE2 was used, forskolin (10 μM), PGE_2_ (1 μM), or control was added into solution. Stimulated duodenal epithelial cellular bicarbonate secretion was to stimulated peak pHi value minus basal pHi value and expressed as ∆pHi.

### Statistics

Statistical analysis was processed by using the SPSS PC statistic package. All results are expressed as means ± standard errors (SE). Data were analyzed by one-way analysis of variance (ANOVA) followed by Newman-Keul’s *post-hoc* test or, when appropriate, by the two-tailed student t tests. *P* < 0.05 was considered statistically significant.

## Results

### Effect of *H. pylori* infection on CFTR, SLC26A6 and TGFβ expressions in murine duodenal mucosa and TGFβ level in murine serum

**Fig. 1:**
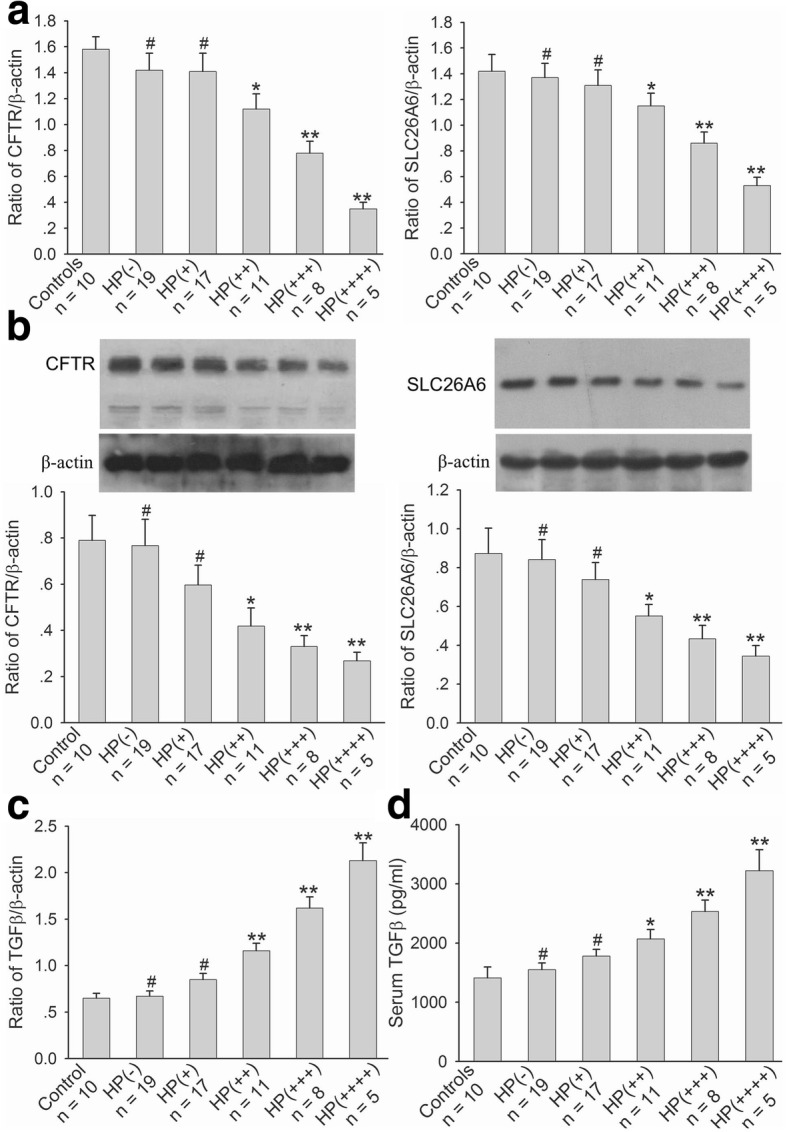
Effect of *H. pylori* infection on CFTR and SLC26A6 mRNA and protein expressions and TGFβ mRNA expression in murine duodenal mucosa and murine serum TGFβ level. Murine *H. pylori-*infected model was established as described in experimental procedures. **a**: Effect of *H. pylori* infection on CFTR and SLC26A6 mRNA expressions in murine duodenal mucosa. **b**: Effect of *H. pylori* infection on CFTR and SLC26A6 protein expressions in murine duodenal mucosa. Upper panels are representative blots and lower panels are the comparisons of expression levels between groups. **c**: Effect of *H. pylori* infection on TGFβ mRNA expression in murine duodenal mucosa. **d**: Effect of *H. pylori* infection on murine serum TGFβ level. Values are mean ± SE in each series. Global *P* < 0.01; ^#^*P* > 0.05, *P < 0.05, **P < 0.01 compared to controls

We first established *H. pylori* infection model in mice. Among 60 experimental mice, 19 (31.67%) were *H. pylori* (−), 17 (28.33%) were *H. pylori* (+), 11 (18.33%) were *H. pylori* (++), 8 (13.33%) were *H. pylori* (+++), and 5 (8.33%) were *H. pylori* (++++). The results from PCR and western blot analyses showed that the mRNA and protein expressions of duodenal mucosal CFTR and SLC26A6 in the mice with *H. pylori* (−) and (+) were not altered in comparison with controls, but there were markedly decrease in the mice with *H. pylori* (++), (+++), and (++++). The mRNA and protein expressions of duodenal mucosal CFTR and SLC26A6 were decreased with the severity of *H. pylori* infection (Fig. 1a and b). The further results showed that duodenal mucosal TGFβ mRNA expression in the mice with *H. pylori* (−) and (+) was not altered in comparison with controls and there were markedly increase in the mice with *H. pylori* (++), (+++), and (++++). The duodenal mucosal TGFβ mRNA expression was increased with the severity of *H. pylori* infection (Fig. 1c). *H. pylori* infection in the mice also induced serum TGFβ concentration increase. The change of serum TGFβ level was in consistent with the change of TGFβ expression in duodenal mucosa and serum TGFβ level was also increased with the severity of *H. pylori* infection (Fig. 1d). These results demonstrate that *H. pylori* infection decreases duodenal mucosal CFTR and SLC26A6 expressions and increases duodenal mucosal TGFβ expression, implying that duodenal mucosal CFTR and SLC26A6 decreases may be related to duodenal mucosal TGFβ increase.

### Effect of *H. pylori* infection on CFTR and SLC26A6 expressions and TGFβ production in duodenal epithelial cells

**Fig. 2:**
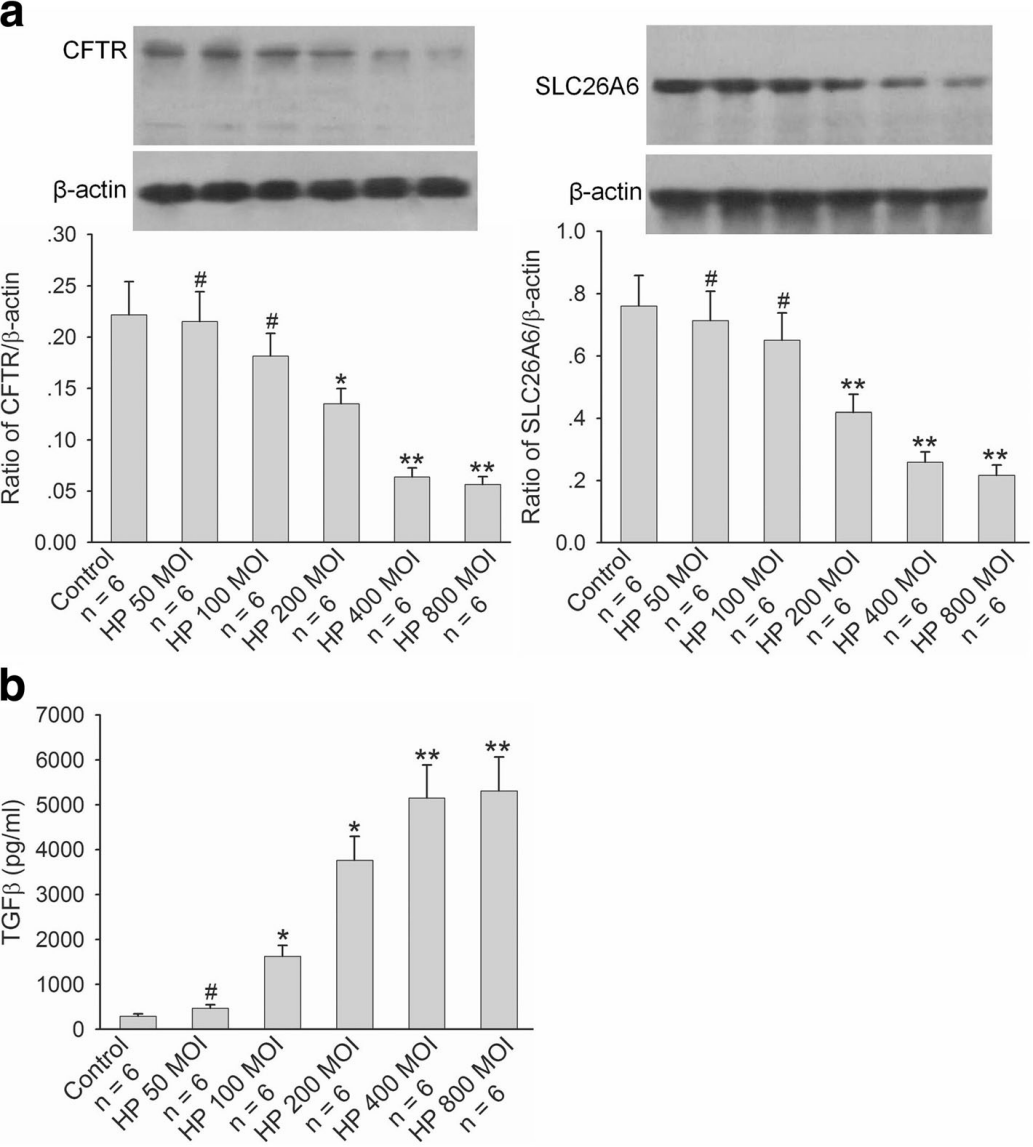
Effect of *H. pylori* infection on CFTR and SLC26A6 protein expressions and TGFβ production in SCBN cells. SCBN cells were treated with different MOI of *H. pylori* for 24 h as described in experimental procedures. **a**: Effect of *H. pylori* infection on CFTR and SLC26A6 protein expressions in SCBN cells. Upper panels are representative blots and lower panels are the comparisons of expression levels between groups. **b**: Effect of *H. pylori* infection on TGFβ production in SCBN cells. Values are mean ± SE in each series. Global *P* < 0.01; ^#^*P* > 0.05, *P < 0.05, **P < 0.01 compared to controls

**Fig. 3:**
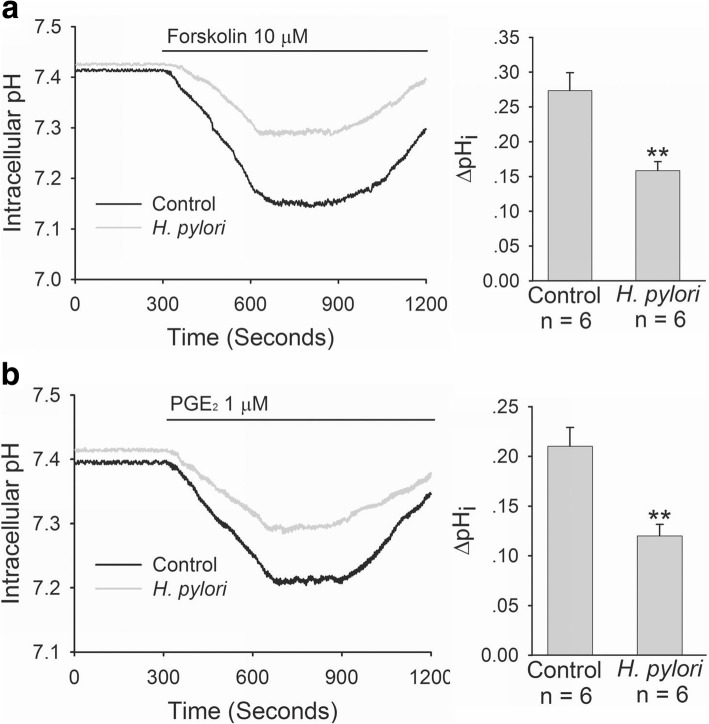
Effect of *H. pylori* infection on forskolin- and PGE_2_-stimulated bicarbonate secretion in SCBN cells. SCBN cells were treated with *H. pylori* at a MOI value of 400 for 24 h. The measurement of bicarbonate secretion in SCBN cells was performed as described in experimental procedures. **a**: Effect of *H. pylori* infection on forskolin- stimulated bicarbonate secretion in SCBN cells. Left panel is time course of change of forskolin-stimulated intracellular pH (pHi) in SCBN cells. Right panel is the comparison of ∆pHi. **b**: Effect of *H. pylori* infection on PGE_2_-stimulated bicarbonate secretion in SCBN cells. Left panel is time course of change of PGE_2_-stimulated intracellular pH (pHi) in SCBN cells. Right panel is the comparison of ∆pHi. Values are mean ± SE in each series. **P < 0.01 compared to controls

**Fig. 4:**
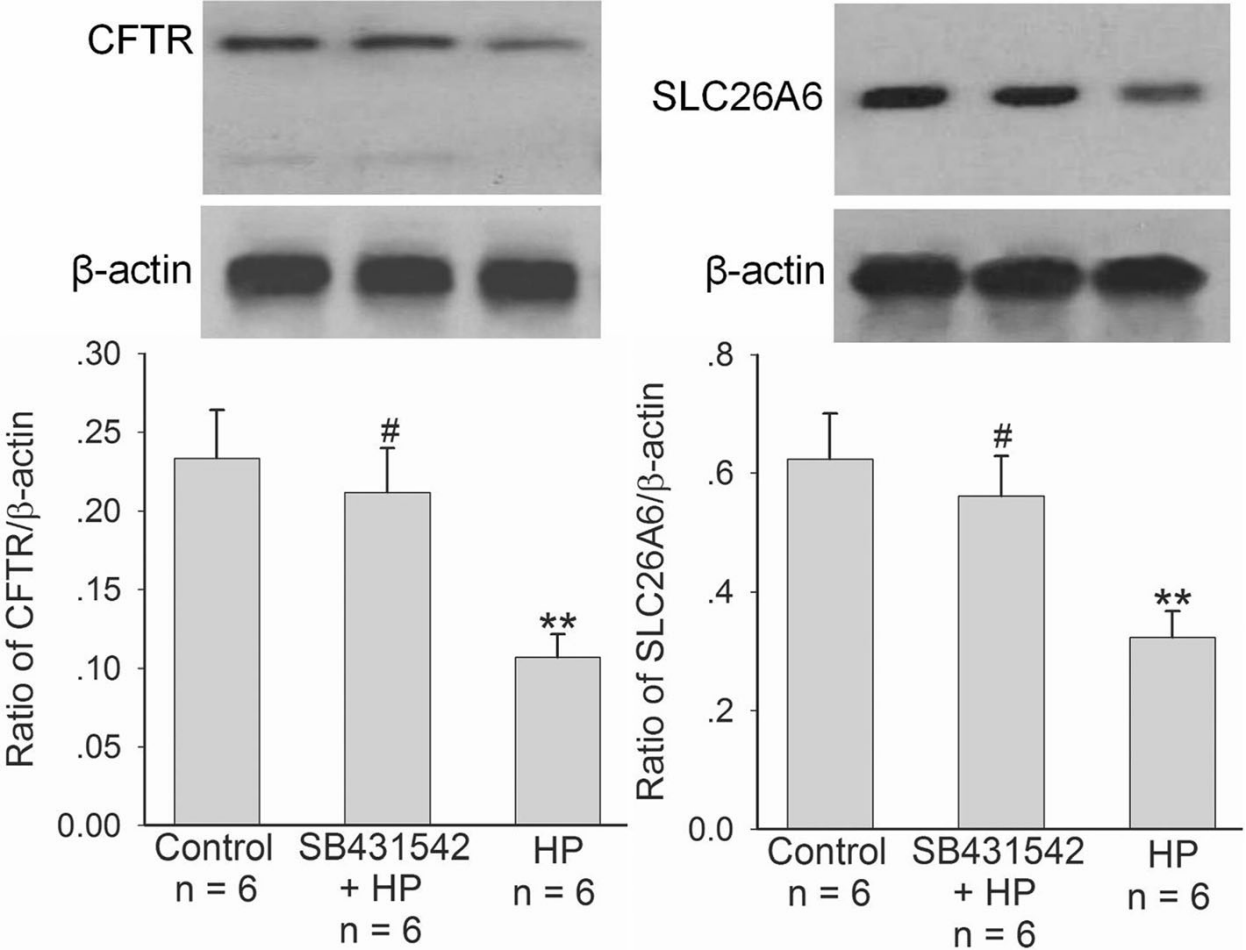
Effect of TGFβ inhibition on *H. pylori-*induced CFTR and SLC26A6 protein expression decreases in SCBN cells. SCBN cells were treated with *H. pylori* at a MOI value of 400 for 24 h. TGFβ inhibitor SB431542 (10 μM) was added at 10 min before *H. pylori.* Upper panels are representative blots and lower panels are the comparisons of expression levels between groups. Values are mean ± SE in each series. ^#^*P* > 0.05, **P < 0.01 compared to controls

We further selected human duodenal epithelial cells, SCBN, to do experiments and investigated the effect of *H. pylori* on CFTR and SLC26A6 expressions and TGFβ production in duodenal epithelial cells. As shown in Fig. 2, after incubation with SCBN cells for 24 h, *H. pylori* induced significant decrease of CFTR and SLC26A6 protein expressions in SCBN cells at a MOI value of 200 in comparison with controls and induced the maximal decrease at a MOI value of 400 (Fig. 2a). In addition, *H. pylori* induced TGFβ production increase in SCBN cells MOI-dependently. Likewise, *H. pylori* induced significant TGFβ production increase in SCBN cells at a MOI value of 200 in comparison with controls and induced the maximal increase at a MOI value of 400 (Fig. 2b). Forskolin, adenylate cyclase activator, is a known CFTR activator and stimulates duodenal mucosal bicarbonate secretion through CFTR, whereas PGE_2_ is believed to stimulate duodenal mucosal bicarbonate secretion mainly through SLC26A6 [20]. The further results showed that forskolin- and PGE_2_-stimulated bicarbonate secretions were markedly decreased in *H. pylori-*infected SCBN cells in comparison with controls (Fig. 3). TGFβ inhibitor SB431542 (10 μM) reversed *H. pylori-*induced CFTR and SLC26A6 protein expression decreases in SCBN cells (Fig. 4). These results indicate that *H. pylori* infection downregulates duodenal epithelial cellular CFTR and SLC26A6 expressions through TGFβ signaling.

### Effect of TGFβ on CFTR and SLC26A6 expression in duodenal epithelial cells

**Fig. 5:**
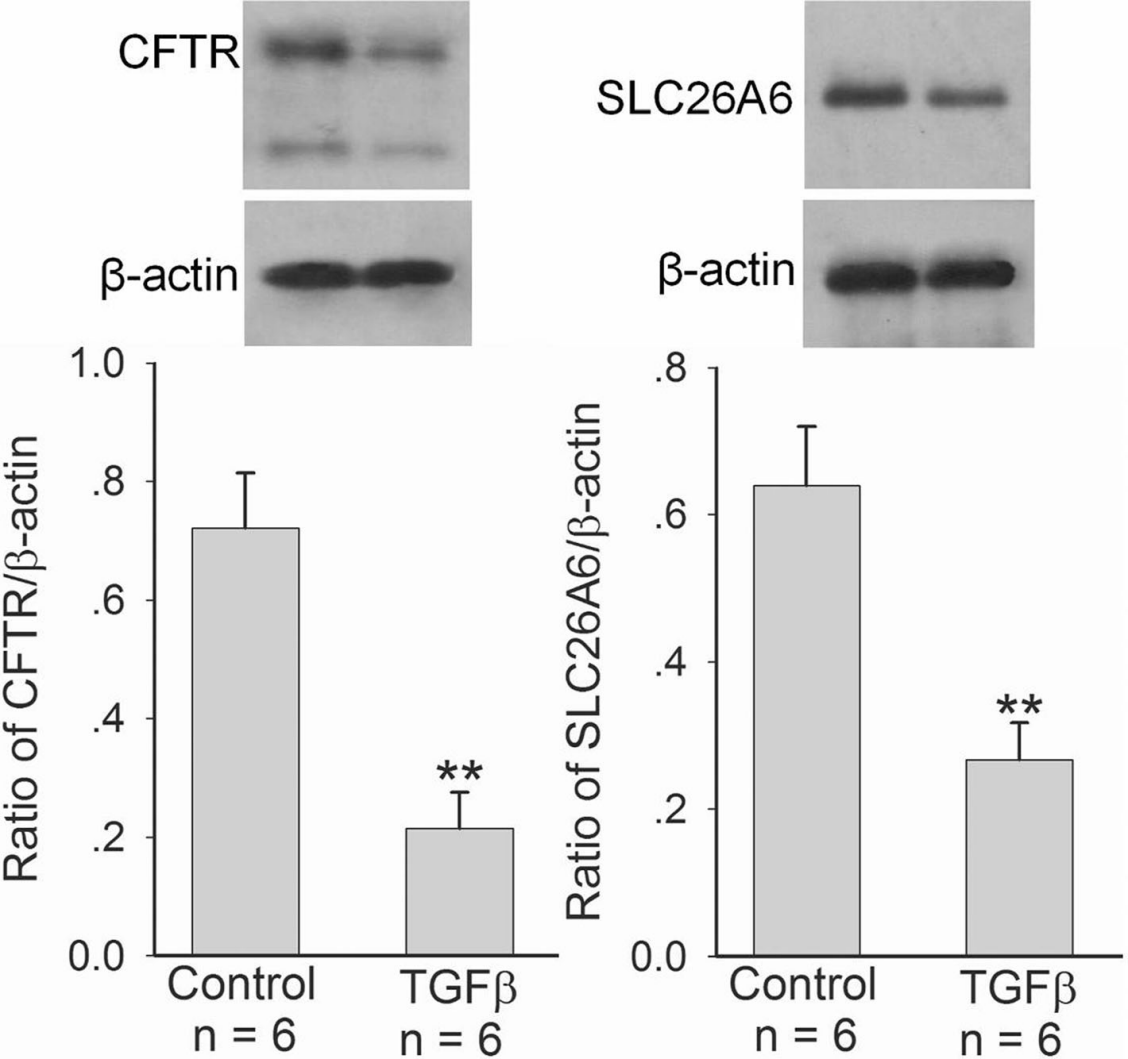
Effect of TGFβ on CFTR and SLC26A6 protein expressions in SCBN cells. SCBN cells were treated with TGFβ (5 ng/ml) for 24 h. Upper panels are representative blots and lower panels are the comparisons of expression levels between groups. Values are mean ± SE in each series. **P < 0.01 compared to controls

**Fig. 6:**
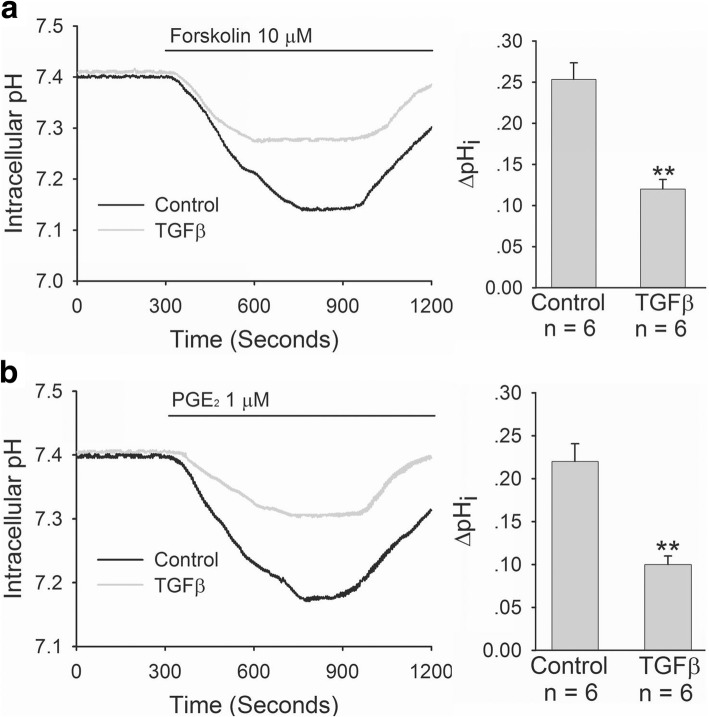
Effect of TGFβ on forskolin- and PGE_2_-stimulated bicarbonate secretions in SCBN cells. SCBN cells were treated with TGFβ (5 ng/ml) for 24 h. The measurement of bicarbonate secretion in SCBN cells was performed as described in experimental procedures. **a**: Effect of TGFβ on forskolin-stimulated bicarbonate secretion in SCBN cells. Left panel is time course of change of forskolin-stimulated intracellular pH (pHi) in SCBN cells. Right panel is the comparison of ∆pHi. **b**: Effect of TGFβ on PGE_2_-stimulated bicarbonate secretion in SCBN cells. Left panel is time course of change of PGE_2_-stimulated intracellular pH (pHi) in SCBN cells. Right panel is the comparison of ∆pHi. Values are mean ± SE in each series. **P < 0.01 compared to controls

**Fig. 7:**
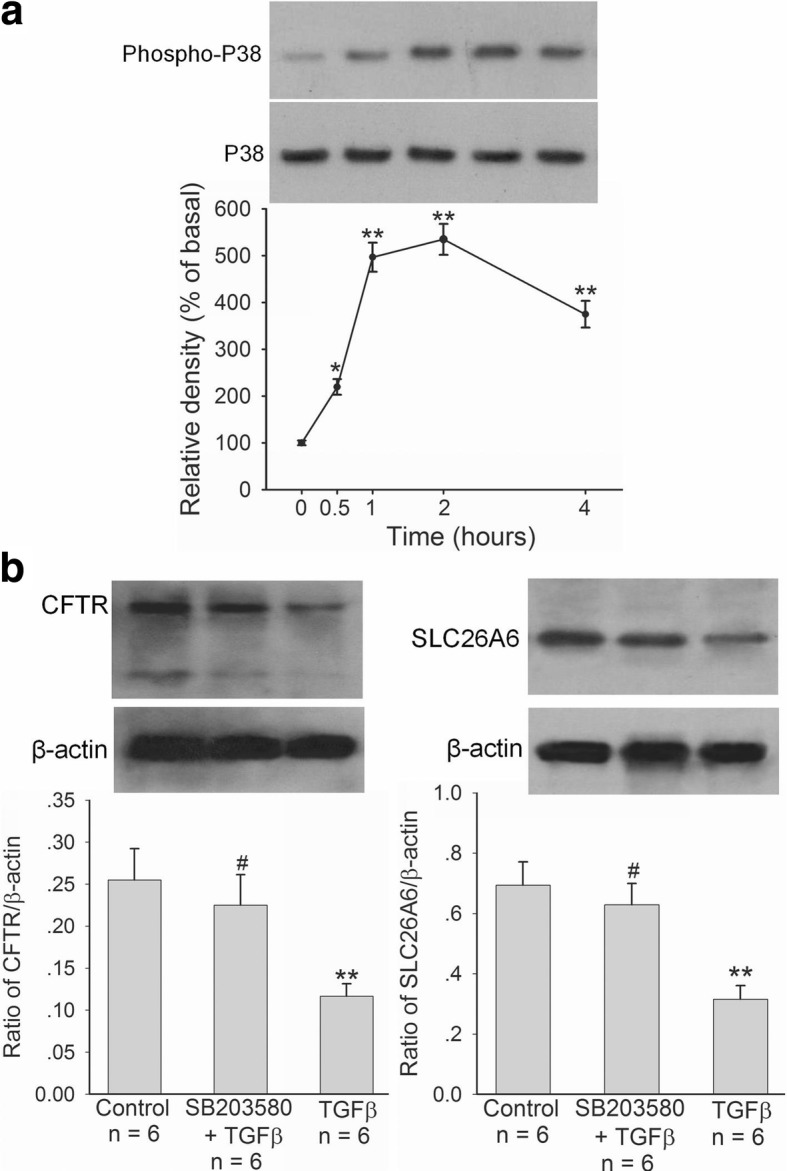
Role of P38 MAPK in TGFβ-induced CFTR and SLC26A6 protein expression decreases in SCBN cells. SCBN cells were treated with TGFβ (5 ng/ml) for 24 h. P38 MAPK inhibitor SB203580 (10 μM) was added at 30 min before TGFβ. **a**: Effect of TGFβ on P38 phosphorylation in SCBN cells. Upper panels are representative blots and lower panels are time course of TGFβ-induced P38 phosphorylation. **b**: Effect of P38 MAPK inhibition on TGFβ-induced CFTR and SLC26A6 protein expression decreases in SCBN cells. Upper panels are representative blots and lower panels are the comparisons of expression levels between groups. Values are mean ± SE in each series. ^#^*P* > 0.05, **P < 0.01 compared to controls

We further investigated whether TGFβ could regulate CFTR and SCL26A6 expressions in duodenal epithelial cells directly. As shown in Figs. 5 and 6, after TGFβ (5 ng/ml) incubated with SCBN cells for 24 h, CFTR and SLC26A6 protein expressions (Fig. 5) and forskolin- and PGE_2_-stimulated bicarbonate secretions (Fig. 6) in SCBN cells were markedly decreased in comparison with controls. The further results showed that TGFβ (5 ng/ml) induced the phosphorylation of P38 mitogen-activated protein kinase (MAPK) in SCBN cells after incubation with SCBN cells for 0.5 h and the maximal response was reached at 2 h (Fig. 7a). P38 MAPK inhibitor SB203580 (10 μM) markedly attenuated TGFβ-induced CFTR and SLC26A6 expression decreases in SCBN cells (Fig. 7b). The results demonstrate that TGFβ downregulates duodenal epithelial cellular CFTR and SLC26A6 expressions through P38 MAPK signaling pathway.

## Discussion

Duodenal mucosal bicarbonate secretion is the most important protective factor against acid-induced duodenal mucosal injury. In this study, we provide evidence that *H. pylori* infection downregulates the expressions of two key bicarbonate transport proteins, CFTR and SLC26A6, in duodenal epithelial cells through TGFβ-mediated P38 MAPK signaling pathway.

*H. pylori* is a Gram-negative bacterium and more than 50% of people were infected with *H. pylori* in the world [21]. It has been demonstrated that *H. pylori* infection is a major cause of duodenal ulcerogenesis [1, 3, 4]. However, the mechanisms whereby *H. pylori* infection causes duodenal ulcerogenesis are not completely understood. Previous studies revealed that *H. pylori* influenced duodenal mucosal bicarbonate secretion which might be involved in the pathogenesis of *H. pylori*-associated duodenal ulcer [7–9]. Our recent study further revealed that *H. pylori* infection downregulated CFTR and SLC26A6 expressions in human duodenal mucosal epithelial cells and the CFTR and SLC26A6 expression decreases were related to the severity and virulent factors of *H. pylori* infection [13]. But, how *H. pylori* influences CFTR and SLC26A6 is not clear. Several pathogenic mechanisms, including *H. pylori* virulence factors and host factors, have been associated with *H. pylori*-induced gastroduodenal diseases. In particular, the immune response against *H. pylori* virulence factors might provide a linkage to the development of gastroduodenal diseases [22–24]. Cytokines have long been considered as the main mediators of the immune response to *H. pylori* infection, which could modulate various intracellular signaling pathways and reprogram host gene expression [25, 26].

In this study, we hypothesized that *H. pylori* might regulate duodenal CFTR and SLC26A6 expressions through cytokines. First, we selected cytotoxin-associated gen A (CagA)- and vacuolating cytotoxin A (VacA)-positive *H. pylori* strain to establish murine *H. pylori* infection model. We found that *H. pylori* infection downregulated duodenal mucosal CFTR and SLC26A6 expressions in mice, which depended on the severity of *H. pylori* infection. On the other hand, *H. pylori* infection induced the increase of serum TGFβ level and duodenal mucosal TGFβ mRNA expression in the mice, which also depended on the severity of *H. pylori* infection. It suggested that a correlation might exist between CFTR and SLC26A6 expressions and TGFβ in duodenal mucosa. We further selected SCBN cells to perform experiments, because SCBN cells are nontransformed duodenal epithelial cells derived from humans and widely used for the study of bicarbonate secretion [14, 27]. The results showed that *H. pylori* increased TGFβ production and decreased CFTR and SLC26A6 expressions in SCBN cells, which depended on multiplicity of infection of *H. pylori.* Moreover, forskolin- and PGE_2_-stimulated bicarbonate secretions were markedly decreased in *H. pylori*-infected SCBN cells in comparison with controls. It has been demonstrated that forskolin stimulates duodenal mucosal bicarbonate secretion through CFTR and PGE_2_-stimulated duodenal mucosal bicarbonate secretion is SLC26A6-dependent [20]. The results further supported that *H. pylori* decreased duodenal epithelial cellular CFTR and SLC26A6 expressions. Further results showed that TGFβ inhibitor SB431542 reversed the *H. pylori*-induced CFTR and SLC26A6 expression decreases. After incubation with SCBN cells for 24 h, TGFβ directly decreased duodenal epithelial cellular CFTR and SLC26A6 expressions by itself and forskolin- and PGE_2_-stimulated bicarbonate secretions were also markedly decreased in TGFβ-treated SCBN cells in comparison with controls. Taken together, these results demonstrate that *H. pylori* downregulates duodenal epithelial cellular CFTR and SLC26A6 expressions through TGFβ signaling pathway.

TGFβ is a multifunctional cytokine that exerts a wide range of biological activities. TGFβ signaling regulates many different cell functions and critical cellular processes. Changes in cellular behavior are governed by activation of TGFβ rectors which triggers subsequent signaling pathways that change gene expression [28, 29]. Previous studies have provided evidence that TGFβ downregulates CFTR expression in polarized T84 human colonocytes and in primary human-airway epithelial cells [30–32]. As a cytokine, TGFβ can be induced through a number of cell types, such as macrophages and lymphocytes. *H. pylori* infection can cause the activation of immune cells, including macrophages, T cells, and B cells, leading to the production of cytokines. Previous studied have revealed that serum TGFβ level is elevated in patients with *H. pylori*-associated gastritis and peptic ulcers, in comparison with *H. pylori*-negative patients [33]. TGFβ mRNA expression is significantly increased in gastric mucosal biopsies of *H. pylori*-infected patients in comparison with *H. pylori*-uninfected patients [34]. In one study, *H. pylori*-secreted soluable proteins stimulated TGFβ production in gastric and colonic epithelial cells [35]. These studies indicate that *H. pylori* infection induces TGFβ production increase and TGFβ signaling might be involved in the pathogenesis of *H. pylori*-associated gastroduodenal diseases. In this study, our results showed that *H. pylori* infection increased TGFβ production and decreased CFTR and SLC26A6 expressions in duodenal epithelial cells. TGFβ inhibition reversed *H. pylori* infection-induced CFTR and SLC26A6 expression decreases, which demonstrates that *H. pylori* infection downregulates duodenal mucosal epithelial cellular CFTR and SLC26A6 expressions through TGFβ signaling, revealing the potential etiological role of TGFβ in *H. pylori*-associated duodenal ulcer.

How does TGFβ regulate duodenal mucosal epithelial cellular CFTR and SLC26A6 expressions? TGFβ is a complex signaling molecule that is activated when TGFβ binds to the TGFβ receptors, then leading to the activation and phosphorylation of the downstream mediators Smad proteins and regulating the expression of target genes in cooperation with other transcription factors, co-activators and co-repressors [28, 36]. In addition to Smad-dependent signaling, the binding of TGFβ to its receptors also activates many noncanonical signaling pathways, including p38 MAPK, external signal-regulated kinase (ERK), and c-Jun N-terminal kinase (JNK) signaling pathways [37, 38]. In this study, our results showed that TGFβ induced the phosphorylation of P38 MAPK in SCBN cells and the inhibition of P38 MAPK reversed the TGFβ-induced CFTR and SLC26A6 expression decreases in SCBN cells, which demonstrates that TGFβ downregulates CFTR and SLC26A6 expressions in duodenal epithelial cells through P38 MAPK signaling pathway.

## Conclusions

The findings from this study demonstrate that *H. pylori* infection downregulates duodenal epithelial cellular CFTR and SLC26A6 expressions through TGFβ-mediated P38 MAPK signaling pathway. CFTR and SLC26A6 are the two key bicarbonate transporters responsible for duodenal mucosal bicarbonate secretion. This study clarifies the mechanism whereby *H. pylori* infection induces duodenal epithelial cellular CFTR and SCL26A6 expression decreases and contributes to further elucidating the pathogenesis of *H. pylori-*associated duodenal ulcer.

## Abbreviations

BCECF-AM: 2′,7′-bis(2-carboxyethyl)-5(6)-carboxy-fluorescein acetoxymethyl ester
CagA: cytotoxin-associated gen A
CFTR: cystic fibrosis transmembrane conductance regulator
*H. pylori*: Helicobacter pylori
MAPK: mitogen-activated protein kinase
PGE_2_: Prostaglandin E_2_
pHi: intracellular pH
SLC26A6: solute linked carrier 26 gene family A6
TGFβ: transforming growth factor β
VacA: vacuolating cytotoxin A
